# Supplementary material for: Heat adaptation of phage T7 under an extended genetic code
Source: Virus Evol. 2021 Dec 1;7(2):veab100. doi: 10.1093/ve/veab100 (PMC8923235; doi:10.1093/ve/veab100)
Supplement: veab100_Supp [file veab100_supp.zip › Sequencing_Analysis.zip!/Sequencing_Analysis/S3/evidence/JC_SIDE_1_54.html]

BRESEQ :: Evidence


| New junction evidence | | | | | | | | | | | |
| --- | --- | --- | --- | --- | --- | --- | --- | --- | --- | --- | --- |
|  | | seq id | position | reads (cov) | reads (cov) | score | skew | freq | annotation | gene | product |
| \* | ? | NC\_001604.1 | = 19585 | 1527 (0.890) | 42 (0.020) | 37/586 | 27.2 | 2.7% | intergenic (–/–) | *–/–* | –/– |
|  | ? | NC\_001604.1 | 19598 = | 1506 (0.900) |  |  |  |  | intergenic (–/–) | *–/–* | –/– |
| Rejected: Coverage evenness skew score above cutoff. | | | | | | | | | | | |
| Rejected: Frequency below/above cutoff threshold. | | | | | | | | | | | |

Only 100 of 1570 total aligned reads displayed.  

`GTACATTCTGGACTCTACCGGAAAACATGTAGCGTACTTCGCGTGGTGCGTAAGCTGTGACATTCACCACAAAGGAGACATTCTGGATGTAACGTCCGTTGTCATTAATCCTGAGGCAGACTCTAAGGGCTTACAGCGATTCCTAGCGAAACGCTTTAAGTACCTTGCGGAACTCCACGATTGCGATTGGGTGTCTCGTTGTAAGCATGAAGGCGAGACAATGCGTGTATACTTTAAGGAGGTATAAGTTATGGGTAAGAAAGTTAAGAAGGCCGTGAAGAAAGTCACCAAGTCCGTTAAGAAAGTCGTTAAGGAAGGGGCTCGTCCGGTTAAACAGGTTGCTGGCGGTCTAGCTGGTCTGGCTGGTGGTACTGGTGAAGCACAGATGGTGGAAGTACCACAAGCTGCCGCACAGATTGTTGACGTACCTGAGAAAGAGGTTTCCACTGAGGACGAAGCACAGACAGAAAGCGGACGCAAGAAAGCTCGTGCTGGCGGTAAGAAATCCTTGAGTGTAGCCCGTAGCTCCGGTGGCGGTATCAACATTTAATCAGGAGGTTATCGTGGAAGACTGCATTGAATGGACCGGAGGTGTCAACTCTAAGGGTTATGG  >  NC_001604.1/19285‑19897`  

                                                                                                                                                                                                                                                                                                            `|`                                                                                                                                                                                                                                                                                                                          
   `cATTCTGGACTCTACCGGAAAACATGTAGCGTACTTCGCGTGGTGCGTAAGCTGTGACATTCACCACAAAGGAGACATTCTGGATGTAACGTCCGTTGTCATTAATCCTGAGGCAGACTCTAAGGGATTACAGCGATTCCTAGCGAAACGCTTTAAGTACCTTGCGGAACTTCACGATTGCGATTGGGTGTCTCGTTGTAAGCATGAAGGCGAGACAATGCGTGTATACTTTAAGGAGGTATAAGTTATGGGTAAGAAAGTTAAGAAGGCCGTGAAGAAAGTCCCCAAGTCCGTTAAGaaa                                                                                                                                                                                                                                                                                                                       >  1:119292/1‑301 (MQ=255)`  
    `aTTCTTGACTCCACCGGAAAACATTGAGCGTACTTGGCGTGGGGCGTAAGACGTGACATTAACCACAAAGGAGTCATTCTGGATGTAACGTCCGTTGTTATTAATCCTGAGGTAGACTCTAAGGGCTTACAGCTATTCCTGACGAAACGCTTTAAGGACCTGGCGGAACTCCACGATTGCGATTGGTTGTCTCGTTGTAAGCATGAAGGCGAGACAATGCGTGTATACTTTAAGGAGGTATAAGTTATGGGTAAGAAAGTTAAGAAGGCCGTGAAGAAAGTCACCAAGTCCGTTAAGAAAg                                                                                                                                                                                                                                                                                                                      <  1:347703/301‑1 (MQ=255)`  
            `ctctACCGGAAAACATGTAGCGTACTTCGCGTGGTGCGTAAGCTGTGACATTCACCACAAAGGAGACATTCTGGATGTAACGTCCGTTGTCATTAATCCTGAGGCAGACTCTAAGGGCTTACAGCGATTCCTAGCGAAACGCTTTAAGTACCTTGCGGAACTCCACGATTGCGATTGGGTGTCTCGTTGTAAGCATGAAGGCGAGACAATGCGTGTATACTTTAAGGAGGTATAAGTTATGGGTAAGAAAGTTAAGAAGGCCGTGAAGAAAGTCACCAAGTCCGTTAAGAAAGTCGTTaag                                                                                                                                                                                                                                                                                                              <  1:881/301‑1 (MQ=255)`  
               `tACCGGAAAACATGTAGCGTACTTCGCGTGGTGCGTAAGCTGTGACATTCACCACAAAGGAGACATTCTGGATGTAACGTCCGTTGTCATTAATCCTGAGGCAGACTCTAAGGGCTTACAGCGATTCCTAGCGAAACGCTTTAAGTACCTTGCGGAACTCCACGATTGCGATTGGGTGTCTCGTTGTAAGCATGAAGGCGAGACAATGCGTGTATACTTTAAGGAGGTATAAGTTATGGGTAAGAAAGTTAAGAAGGCCGTGAAGAAAGTCACCAAGTCCGTTAAGAAAGTCGTTaaggaa                                                                                                                                                                                                                                                                                                           <  1:34394/301‑1 (MQ=255)`  
                              `aGCGTACTTCGCGTGGTGCGTAAGCTGTGACATTCACCACAAAGGAGACATTCTGGATGTAACGTCCGTTGTCATTAATCCTGAGGCAGACTCTAAGGTCTTACAGCGATTCCTAGCGAACCGCTTTAAGTACCTTGCGGAACTCCACGATTGCGATTGGGTGTCTCGTTGTAAGCATGAAGGCGAGACAATGCGTGTATACTTTAAGGAGGTATAAGTTATGGGTAAGAAAGTTAAGAAGGCCGTGAAGAAAGTCACCAAGTCCGTTAAGAAAGTCGTTaaggaaggctgtctcttatac                                                                                                                                                                                                                                                                                            >  1:123749/1‑288 (MQ=255)`  
                               `gCGTACTTCTCGTGGTGCGTAAGCTGTGACATTCACCACAAAGGAGACATTCTGGATGTAACGTCCGTTGTCATTAATCCTGAGGCAGACTCTAAGGGCTTACAGCGATTCCTAGCGAAACGCTTTAAGTACCTTGCGGAACTCCACGATTGCGATTGGGTGTCTCGTTGTAAGCATGAAGGCGAGACAATGCGTGTATACTTTAAGGAGGTATAAGTTATGGGTAAGAAAGTTAAGAAGGCCGTGAAGAAAGTCACCAAGTCCGTTAAGAAAGTCGTTAAGGAAGGGGCTctgtctctta                                                                                                                                                                                                                                                                                           >  1:247671/1‑292 (MQ=255)`  
                                    `cTTCGCGTGGTGCGTAAGCTGTGACATTCACCACAAAGGAGACATTCTGGATGTAACGTCCGTTGTCATTAATCCTGAGGCAGACTCTAAGGGCTTACAGCGATTCCTAGCGAAACGCTTTAAGTACCTTGCGGAACTCCACGATTGCGATTGGGTGTCTCGTTGTAAGCATGAAGGCGAGACAATGCGTGTATACTTCAAGGAGGTATAAGTTATGGGTAAGAAAGTTAAGAAGGCCGTGAAGAAAGTCACCAAGTCCGTTAAGAAAGTCGTTAAGGAAGGGGCTCGTCCGGTTAAACAg                                                                                                                                                                                                                                                                                      >  1:274193/1‑301 (MQ=255)`  
                                    `cTTCGCGTGGTGCGTAAGCTGTGACAGTGACCACAAAGGCGACATTCTGGATGTAACGTCCGTTGTCAGTAATCCTGAGGCAGACTCTAAGGGCTTACAGCGATTCCTAGCGAAACGCTTTAAGAACCTTGCGGAACTCCACGATTGCGATTGGGTGTCTCGTTGTAAGCATGAAGGCGAGACAATGCGTGTATACTTTAAGGAGGTATAAGTTATGGGTAAGAAAGTTAAGAAGGCCGTGAAGAAAGTCACCAAGTCCGTTAAGAAAGTCGTTAAGGAAGGGGCTCGTCCGGTTAAACAg                                                                                                                                                                                                                                                                                      <  1:182115/301‑1 (MQ=255)`  
                                    `cTTCGCGGTGTGCGTAAGCTGTGACATTCACCACAAAGGAGACATTCTGGATGTAACGTCCGTTGTCATTAATCCTGAGGCAGACTCTAAGGGCTTACAGCGATTCCTAGCGAAACGCTTTAAGTACCTTGCGGAACTCCACGATTGCGATTGGGTGTCTCGTTGTAAGCATGAAGGCGAGACAATGCGTGTATACTTTAAGGAGGTATAAGTTATGGGTAAGAAAGTTAAGAAGGCCGTGAAGAAAGTCACCAAGTCCGTTAAGAAAGTCGTTAAGGAAGGGGCTCGTCCGGTTAAACAg                                                                                                                                                                                                                                                                                      <  1:210041/301‑1 (MQ=255)`  
                                     `ttCGCGTGGTGCGTAAGCTGTGACATTCACCACAAAGGAGACATTCTGGATGTAACGTCCGTTGTCATTAATCCTGAGGCAGACTCTAAGGGCTTACAGCGATTCCTAGCGAAACGCTTTAAGTACCTTGCGGAACTCCACGATTGCGATTGGGTGTCTCGTTGTAAGCATGAAGGCGAGACAATGCGTGTATACTTTAAGGAGGTATAAGTTATGGGTAAGAAAGTTAAGAAGGCCGTGAAGAAAGTCACCAAGTCCGTTAAGAAAGTCGTTAAGGAAGGGGCTCGTCCGGTTAAACAgg                                                                                                                                                                                                                                                                                     <  1:244988/301‑1 (MQ=255)`  
                                               `gCGTAAGCTGTGACATTCACCACAAAGGAGACATTCTGGATGTAACGTCCGTTGTCATTAATCCTGAGGCAGACTCTAAGGGCTTACAGCGATTCCTAGCGAAACGCTTTAAGTACCTTGCGGAACTCCACGATTGCGATTGGGTGTCTCGTTGTAAGCATGACGGCGAGACAATGCGTATATACTTTAAGGAGGTATAAGTTATGGGTAAGAAAGTTAAGAAGGCCGTGAAGAAAGTCACCAAGTC‑‑‑‑‑‑‑‑‑‑‑‑CGTTAAGGAAGGGGCTCGTCCGGTTAAACAGGTTGCTGGCGGTCTAGCTGGTct                                                                                                                                                                                                                                                               >  1:268397/1‑301 (MQ=255)`  
                                                        `gtgCCATTCACCACAAAGGAGACATTCTGGATGTAACGTCCGTTGTCATTAATCCTGAGGCAGACTCTAAGGGCTTACAGCGATTCCTAGCGAAACGCTTTAAGTACCTTGCGGAACTCCACGATTGCGATTGGGTGTCTCGTTGTAAGCATGAAGGCGAGACAATGCGTGTATACTTTAAGGAGGTATAAGTTATGGGTAAGAAAGTTAAGAAGGCCGTGAAGAAAGTCACCAAGTCCGTTAAGAAAGTCGTTAAGGAAGGGGCTCGTCCGGTTAAACAGGTTGCTGGCGGTCTAGCtag                                                                                                                                                                                                                                                                  >  1:257136/1‑299 (MQ=255)`  
                                                              `ttCACCACAAAGGAGACATTCTGGATGTAACGTCCGTTGTCATTAATCCTGAGGCAGACTCTAAGGGCTTACAGCGATTCCTAGCGAAACGCTTTAAGTACCTTGCGGAACTCCACGATTGCGATTGGGTGTCTCGTTGTAAGCATGAAGGCGAGACAATGCGTGTATACTTTAAGGAGGTATAAGTTATGGGTAAGAAAGTTAAGAAGGCCGTGAAGAAAGTCACCAAGTCCGTTAAGAAAGTCGTTAAGGAAGGGGCTCGTCCGGTTAAACAGGTTGCTGGCGGTCTAGCTGGTctggc                                                                                                                                                                                                                                                            <  1:321923/301‑1 (MQ=255)`  
                                                               `tCACCACAAAGGAGACATTCTGGATGTAACGTTCGTTGTCATTAATCCTGAGGCAGACTCTAAGGGCTGACAGCGATTCCTAGCGAAACGCTTTAAGTACCTTGCGGAACTCCACGATTGCGATTGGGTGTCTCGTTGTAAGCATGAAGGCGAGACAATGCGTGTATACTTTAAGGAGGTATAAGTTATGGGTAAGAAAGTTAAGAAGGCCGTGAAGAAAGTCACCAAGTCCGTTAAGAAAGTCGTTAAGGAAGGGGCTCGTCCGGTTAAACAGGTTGCTGGCGGTCTAGCTGGTctggct                                                                                                                                                                                                                                                           <  1:19466/301‑1 (MQ=255)`  
                                                                  `ccacAAAGGAGACATTCTGGATGTAACGTCCGTTGTCATTAATCCTGAGGCAGACTCTAAGGGCTTACAGCGATTCCTAGCGAAACGCTTTAAGTACCTTGCGGAACTCCACGATTGCGATTGGGTGTCTCGTTGTAAGCATGAAGGCGAGACAATGCGTGTATACTTTAAGGAGGTATAAGTTATGGGTAAGAAAGTTAAGAAGGCCGTGAAGAAAGTCACCAAGTCCGTTAAGAAAGTCGTTAAGGAAGGGGCTCGTCCGGTTAAACAGGTTGCTGGCGGTCTAGCTGGTctggctggg                                                                                                                                                                                                                                                        >  1:212774/1‑300 (MQ=255)`  
                                                                   `aataaaGGTGACATTTTGTATGTATCATCTGTTGTTTTTAATACTGAGTCATACTGTAAGTGCTTACAGCTTTTCCTAGCGAAACGCTTTAAGTCCATTGCGAAACACTTAGTTTGCGATTGGGTGGCTCGTTGTAAGCATGAAGGCGAGTCAATGCTTGTATACTTTACGGTGGTATAAGTTATGGGTAAGAAATTTAAGAAGGCCGTGAAGAAAGTCACCAAGTCCGTTAAGAAAGTCGTTAAGGAAGGGGCTCGTCCGGTTAAACAGGTTGATGGCGGTCTAGCTGGtggtactggtg                                                                                                                                                                                                                                                       <  1:197152/298‑11 (MQ=255)`  
                                                                        `aGGAGACATTCTGGATGTAACGTCCGTTGTCATTAATCCTGAGGCAGACTCTAAGGGCTTACAGCGATTCCTAGCGAAACGCTTTAAGTACCTTGCGGAACTCCACGATTGCGATTGGGTGTCTCGTTGTAAGCATGAAGGCGAGACAATGCGTGTATACTTTAAGGATGTATAAGTTATGGGTAAGAAAGTTAAGAAGGCCGTGAAGAAAGTCACCAAGTCCGTTAAGAAAGTCGTTAAGGAAGGGGCTCGTCCGGTTAAACAGGTTGCTGGCGGTCTATCTGGTCTGGCTGGTGGTACt                                                                                                                                                                                                                                                  <  1:96506/301‑1 (MQ=255)`  
                                                                         `ggAGACATTCTGGATGTAACGTCCGTTGTCATTAATCCTGAGGCAGACTCTAAGGGCTTACAGCGATTCCTAGCGAAACGCTTTAAGTACCTTGCGGAACTCCACGATTGCGATTGGGTGTCTCGTTGTAAGCATGAAGGCGAGACAATGCGTGTATACTTTAAGGAGGTATAAGTTATGGGTAAGAAAGTTAAGAAGGCCGTGAAGAAAGTCACCAAGTCCGTTAAGAAAGTCGTTAAGGAAGGGGCTCGTCCGGTTAAACAGGTTGCTGGCGGTCTAGCTGGTCTGGctgctgtctctt                                                                                                                                                                                                                                                 >  1:90813/1‑295 (MQ=255)`  
                                                                                `ttCTGGATGTAACGTCCGTTGTCATTAATCTTGAGGCAGACTCTAAGGGCTTACAGCGATTCCTAGCGAAACGCTTTAAGTACCTTGCGGAACTCCACGATTGCGATTGGGTGTCTCGTTGTAAGCATGAAGGCGAGACAATGCGTGTATACTTTAAGGAGGTATAAGTTATGGGTAAGAAAGTTAAGAAGGCCGTGAAGAAAGTCACCACGTCCGTTAAGAAAGTCGTTAAGGAAGGGGCTCGTCCGGTTAAACAGGTTGCTGGCGGTCTAGCTGGTcctgtctcttatacacatctccg                                                                                                                                                                                                                                          >  1:324438/1‑279 (MQ=255)`  
                                                                                  `cTGGATGTAACGTCCGTTGTAATTAATCCTGAGGCAGACTCTAAGGGCTTACAGCGATTCCTAGCGAAACGCTTTAAGTACCTTGCGGAACTCCACGATTGCGATTGGGTG‑‑TCGTTGTAAGCATGAAGGCGAGACAATGCGTGTATACTTTAAGGAGGTATAAGTTATGGGTAAGAAAGTTAAGAAGGCCGTGAAGAAAGTCACCAAGTCCGTTAAGAAAGTCGTTAAGGAAGGGGCTCGTCCGGTTAAACAGGTTGCTGGCGGTCTAGCTGGTCTGGCTGGTGGTACTGGTGAAGCACAg                                                                                                                                                                                                                                      <  1:55303/301‑1 (MQ=255)`  
                                                                                     `aagagacagTCCGTTGTCATTAATCCTGAGGCAGACTCTAAGGGCTTACAGCGATTCCTAGCGAAACGCTTTAAGTACCTTGCGGAACTCCACGATTGCGATTGGGTGTCTCGTTGTAAGCATGAAGGCGAGACAATGCGTGTATACTTTAAGGAGGTATAAGTTATGGGTAAGAAAGTTAAGAAGGCCGTGAAGAAAGTCACCAAGTCCGTTAAGAAAGTCGTTAAGGAAGGGGCTCGTCCGGTTAAACAGGTTGCTGGCGGTCTAGCTGGTCTGGCTGGTGGTACTGGTGAAGCACAGa                                                                                                                                                                                                                                     <  1:206661/293‑1 (MQ=255)`  
                                                                                        `gTAACGTCCGTTGTCATTAATCCTGAGGCAGACTCTAAGGGCTTACAGCGATTCCTAGCGAAACGCTTTAAGTACCTTGCGGAACTCCACGATTGCGATTGGGTGTCTCGTTGTAAGCATGAAGGCGAGACAATGCGTGTATACTTTAAGGAGGTATAAGTTATGGGTAAGAAAGTTAAGAAGGCCGTGAAGAAAGTCACCAAGTCCGTTAAGAAAGTCGTTAAGGAAGGGGCTCGTCCGGTTAAACAGGTTGCTGGCGGTCTAGCTGGTCTGGCTGGTGGTACTGGTGAAGCACAGAtgg                                                                                                                                                                                                                                  >  1:264825/1‑301 (MQ=255)`  
                                                                                          `tataagagacagcATTAATCCTGAGGCAGACTCTAAGGGCTTACAGCGATTCCTAGCGAAACGCTTTAAGTACCTTGCGGAACTCCACGATTGCGATTGGGTGTCTCGTTGTAAGCATGAAGGCGAGACAATGCGTGTATACTTTAAGGAGGTATAAGTTATGGGTAAGAAAGTTAAGAAGGCCGTGAAGAAAGTCACCAAGTCCGTTAAGAAAGTCGTTAAGGAAGGGGCTCGTCCGGTTAAACAGGTTGCTGGCGGTCTAGCTGGTCTGGCTGGTGGTACTGGTGAAGCACAGAtggtg                                                                                                                                                                                                                                <  1:4446/289‑1 (MQ=255)`  
                                                                                            `cGTCCGTGGTCATTAATCCTGAGGCAGACTCTAAGGGCTTACAGCGATTCCTAGCGAAACGCTTTAAGTACCTTGCGGAACTCCACGATTGCGATTGGGTGTCTCGTTGTAAGCATGAAGGCGAGACAATGCGTGTATACTTTAAGGAGGTATAAGTTATGGGTAAGAAAGTTAAGAAGGCCGTGAAGAAAGTCACCAAGTCCGTTAAGAAAGTCGTTAAGGAAGGGGCTCGTCCGGTTAAACAGGTTGCTGGCGGTCTAGCTGGTCTGGCTGGTGATACTGGTGAAGCACAGATGGTGGa                                                                                                                                                                                                                              <  1:29200/301‑1 (MQ=255)`  
                                                                                               `cctttGTCATTAATCCTGAGGCAGACTCTAAGGGCTTACAGCGATTCCTAGCGAAACGATTTAAGTACCTTGCGGAACTCCACGATTGCGATTGGGTGTCTCGTTGTAAGCATGAAGGCGAGACAATGCGTGTATACTTTAAGGAGGTATAAGTTATGGGTAAGAAAGTTAAGAAGGCCGTGAAGAAAGTCACCAAGTCCGTTAAGAAAGTCGTTAAGGAAGGGGCTCGTCCGGTTAAACAGGTTGCTGGCGGTCTAGCTGGTCTGGCTGGTGGTACTGGTGAAGCACAGATGGTGGAAGt                                                                                                                                                                                                                           <  1:295105/298‑1 (MQ=255)`  
                                                                                                   `cagcATTAATCCTGAGGCAGACTCTAAGGGCTTACAGCGATTCCTAGCGAAACGCTTTAAGTACCTTGCGGAACTCCACGATTGCGATTGGGTGTCTCGTTGTAAGCATGAAGGCGAGACAATGCGTGTATACTTTAAGGAGGTATAAGTTATGGGTAAGAAAGTTAAGAAGGCCGTGAAGAAAGTCACCAAGTCCGTTAAGAAAGTCGTTAAGGAAGGGGCTCGTCCGGTTAAACAGGTTGCTGGCGGTCTAGCTGGTCTGGCTGGTGGTACTGGTGAAGCACAGATGGTGGAAGTACca                                                                                                                                                                                                                       >  1:86361/4‑301 (MQ=255)`  
                                                                                                    `gTCATTAATCCTGAGGCAGACTCTAAGGGCTTACAGCGATTCCTAGCGAAACGCTTTAAGTACCTTGCGGAACTCCACGATTGCGATTGGGTGTCTCGTTGTAAGCATGAAGGCGAGACAATGCGTGTATACTTTAAGGAGGTATAAGTTATGGGTAAGAAAGTTAAGAAGGCCGTGAAGAAAGTCACCAAGTCCGTTAAGAAAGTCGTTAAGGAAGGGGCTCGTCCGGTTAAACAGGTTGCTGGCGGTCTAGCTGGTCTGGCTGGTGGTACTGGTGAAGCACAGAtctgtctcttataca                                                                                                                                                                                                                      >  1:160188/1‑287 (MQ=255)`  
                                                                                                    `gTCATTAAACCGGAGACAGACTCTAAGGGCTTAAAGGGATTCCAAGCGAAACGCTTAAAGTACCTTGCGGAACTCCACGATGGCGATTGGGTGTCTCGTTTTAAGCATGAAGGCGAGACAATGCGTGTATACTTTAAGGAGGTATAAGTTATGGGTAAGAAAGTTAAGAAGGCCGTGAAGAAAGTCACCAAGTCCGTTAAGAAAGTCGTTAAGGAAGGGGCTCGTCCGGTTAAACAGGTTGCTGGCGGTCTAGCTGGTCTGGCTGGTGGTACTGGTGAAGCACAGATGGTGGAAGTACcac                                                                                                                                                                                                                      <  1:149976/301‑1 (MQ=255)`  
                                                                                                      `cATTAATCCTGAGGCAGACTCTAAGGGCTTACAGCGATTCCTAGCGAAACGCTTTAAGTACCTTGCGGAACTCCACGATTGCGATTGGGTGTCTCGTTGTAAGCATGAAGGCGAGACAATGCGTGTATACTTTAAGGAGGTATAAGTTATGGGTAAGAAAGTTAAGAAGGCCGTGAAGAAAGTCACCAAGTCCGTTAAGAAAGTCGTTAAGGAAGGGGCTCGTCCGGTTAAACAGGTTGCTGGCGGTCTAGCTGGTCTGGCTGGTGGTACTGGTGAAGCACAGATGGTGGAAGTACCACaa                                                                                                                                                                                                                    >  1:21957/1‑301 (MQ=255)`  
                                                                                                           `aTCCTGAGGCAGACTCTAAGGGCTTACAGCGATTTCTAGCGAAACGCTTTAAGTACCTTGCGGAACTCCACGATTGCGATTGGGTGTCTCGTTGTAAGCATGAAGGCGAGACAATGCGTGTATACTTTAAGGAGGTATAAGTTATGGGTAAGAAAGTTAAGAAGGCCGTGAAGAAAGTCACCAAGTCCGTTAAGAAAGTCGTTAAGGAAGGGGCTCGTCCGGTTAAACAGGTTGCTGGCGGTCTAGCTGGTCTGGCTGGTGGTACTGGTGAAGCACAGATGGTGGAAGTACCACAAGCtct                                                                                                                                                                                                               >  1:338311/1‑299 (MQ=255)`  
                                                                                                            `tCCTGAGGCAGACTCTAAGGGCTTACAGCGATTCCTAGCGAAACGCTTTAAGTACCTTGCGGAACTCCACGATTGCGATTGGGTGTCTCGTTGTAAGCATGAAGGCGAGACAATGCGTGTATACTTTAAGGAGGTATAAGTTATGGGTAAGAAAGTTAAGAAGGCCGTGAAGAAAGTCACCAAGTCCGTTAAGAAAGTCGTTAAGGAAGGGGCTCGTCCGGTTAAACAGGTTGCTGGCGGTCTAGCTGGGCTGGCTGGTGGTACTGGTGAAGCACAGATGGTGGAAGTACCACAAGCTGcc                                                                                                                                                                                                              >  1:108832/1‑301 (MQ=255)`  
                                                                                                                  `acaggACTCTAAGGGCTTACAGCGATTCCTAGCGAAACGCTTTAAGTACCTTGCGGAACTCCACGATTGCGATTGGGTGTCTCGTTGTAAGCATGAAGGCGAGACAATGCGTGTATACTTTAAGGAGGTATAAGTTATGGGTAAGAAAGTTAAGAAGGCCGTGAAGAAAGTCACCAAGTCCGTTAAGAAAGTCGTTAAGGAAGGGGCTCGTCCGGTTAAACAGGTTGCTGGCGGTCTAGCTGGTCTGGCTGGTGGTACTGGTGAAGCACAGATGGTGGAAGTACCACAAGCTGCCGCACAg                                                                                                                                                                                                        <  1:170814/297‑1 (MQ=255)`  
                                                                                                                      `gACTCTAAGGTCTTACAGCGATTCCTAGCGAAACGCTTTAAGTACCTTGCGTAACTCCACGATTGCGATTGGTTGTCTCGTTGTAAGCATGAAGGCGAGACAATGTGTGTATACTTTACGGAGGTATAAGTTATGGGTAAGCACGTTACGAAGGCCGTGAAGAAAGTCACCAAGTCCGTTAAGAACGTCGTTAAGGAAGGGGCTCGTCCGGTTAATCAGGTTGCTGGCGGTCTAGCTGGTATGTCTGTTGGTACTGTTGAAGCACTGATGGTGGAAGTCCCACAATCTGCAGcacatgttg                                                                                                                                                                                                    >  1:48016/1‑296 (MQ=255)`  
                                                                                                                        `ctctAAGGGCTTACAGCGATTCCTAGCGAAACGCTTTAAGTACCTTGCGGAACTCCACGATTGCGATTGGGTGTCTCGTTGTAAGCATGAAGGCGAGACAATGCGTGTATACTTTAAGGAGGTATAAGTTATGGGTAAGAAAGTTAAGAAGGCCGTGAAGAAAGTCACCAAGTCCGTTAAGAAAGTCGTTAAGGAAGGGGCTCGTCCGGTTAAACAGGTTGCTGGCGGTCTAGCTGGTCTGGCTGGTGGTACTGGTGAAGCACAGATGGTGGAAGTAcctgtctcttatacacatctccga                                                                                                                                                                                                  >  1:154549/1‑279 (MQ=255)`  
                                                                                                                        `ctctAAGGGCTTACAGCGATTCCTAGCGAAACGCTTTAAGTACCTTGCGGAACTCCACGATTGCGATTGGGTGTCTCGTTGTAAGCATGAAGGCGAGACAATGCGTGTATACTTTAAGGAGGTATAAGTTATGGGTAAGAAAGTTAAGAAGGCCGTGAAGAAAGTCACCAAGTCCGTTAAGAAAGTCGTTAAGGAAGGGGCTCGTCCGGTTAAACAGGTTGCTGGCGGTCTAGCTGGTCTGGCTGGTGGTACTGGTGAAGCACAGATGGTGGAAGTACCACAAGCTGCCGCACAGAttgtt                                                                                                                                                                                                  >  1:224624/1‑301 (MQ=255)`  
                                                                                                                        `ctctAAGGGCTTACAGCGATTCCTAGCGAAACGCTTTAAGTACCTTGCGGAACTCCACGATTGCGATTGGGTGTCTCGTTGTAAGCATGAAAGCGAGACAATGCGTGTATACTTTAAGGAGGTATAAGTTATGGGTAAGAAAGTTAAGAAGGCCGTGAAGAAAGTCACCAAGTCCGTTAAGAAAGTCGTTAAGGAAGGGGCTCGTCCGGTTAAACAGGTTGCTGGCGGTCTAGCTGGTCTGGCTGGTGGTACTGGTGAAGCACAGATGGTGGAAGTACCACAAGCTGCCGCACAGAttgtt                                                                                                                                                                                                  >  1:301818/1‑301 (MQ=255)`  
                                                                                                                               `ggCTTACAGCGATTCCTAGCGAAACGCTTTAAGTACCTTGCGGAACTCCACGATTGCGATTGGGTGTCTCGTTGTAAGCATGAAGGCGAGACAATGCGTGTATACTTTAAGGAGGTATAAGTTATGGGTAAGAAAGTTAAGAAGGCCGTGAAGAAAGTCACCAAGTCCGTTAAGAAAGTCGTTAAGGAAGGGGCTCGTCCGGTTAAACAGGTTGCTGGCGGTCTAGCTGGTCTGGCTGGTGGTACTGGTGAAGCACAGATGGTGGAAGTACCACAAGCTGCCGCACAGATTGTTGActgtc                                                                                                                                                                                           >  1:270509/1‑297 (MQ=255)`  
                                                                                                                               `ggCTTACAGCGATTCCTAGCGAAACGCTTTAAGTACCTTGCGGAACTCCACGATTGCGATTGGGTGTCTCGTTGTAAGCATGAAGGCGAGACAATGCGTGTATACTTTAAGGAGGTATAAGTTATGGGTAAGAAAGTTAAGAAGGCCGTGAAGAAAGTCACCAAGTCCGTTAAGAAAGTCGTTAAGGAAGGGGCTCGTCCGGTTAAACAGGTTGCTGGCGGTCTAGCTGGTCTGGCTGGTGGTACTGGTGAAGCACAGATGGTGGAAGTACCACAAGCTGCCGCACAGATTGTTGACGTAc                                                                                                                                                                                           >  1:260602/1‑301 (MQ=255)`  
                                                                                                                               `ggCTTACAGCGATTCCTAGCGAAACGCTTTAAGTACCTTGCGGAACTCCACGATTGCGATTGGGTGTCTCGTTGTAAGCATGAAGGCGAGACAATGCGTGTATACTTTAAGGAGGTATAAGTTATGGGTAAGAAAGTTAAGAAGGCCGTGAAGAAAGTCACCAAGTCCGTTAAGAAAGTCGTTAAGGAAGGGGCTCGTCCGGTTAAAAATGTTGCTGGCGGTCTAGCTGGTCTGGCTGGTGGTACTGGTGAAGCACAGATGGTGGAAGTACCACAAGCTGCCGCACAGAttgtctgtctct                                                                                                                                                                                           >  1:78351/1‑293 (MQ=255)`  
                                                                                                                                  `taaCAGCGTTTCCTAGCGAAACGCTGTAAGTTGCTTGCGGAACTCAACGATTGCGATTGGGTGTCTCGTTGTCAGCATGAAGGAGAGACAATGGGTGTATACTTTAAGGAGGTAGAAGTTATGGGTAAGAAAGTTAAGAATGCCGTGAAGAAAGCCACCAAGTCCGTTAAGTAAGACGTTAAGGAAGGGGCTCGTCCGGTAAAACAGGTTGCTGGAGGTCTAGCTTGTCTGGCTGGTGGTACTGGTGAAGCACAGATGGTGGAAGTACCACAAGCTGCCGCACAGATTGTTGACGTACCTg                                                                                                                                                                                        <  1:184670/299‑1 (MQ=255)`  
                                                                                                                                     `cAGCGATTCCTAGCGAAACGCTTTAAGTACCTTGCGGAACTCCACGATTGCGATTGGGTGCCTCGTTGTAAGCATGAAGGCGAGACAATGCGTGTATACTTTAGGGAGGTATAAGTTATGGGTAAGAAAGTTAGGAAGGCCGTGAAGAAAGTCACCAAGTCCGTTAAGAAAGTCGTTAAGGAAGGGGCTCGTCGGGTTAAACAGGTTGCTGGCGGTCTAGCTGGTCTGGCTGGTGGTACTGGTGAGGCACAGAGGGTGGAAGTACCAAAGGCTGCGGCAAGGATTGTTGCCGTACCTggga                                                                                                                                                                                     >  1:277554/1‑301 (MQ=255)`  
                                                                                                                                       `gCGATTCCTAGCGAAACGCTTGAAGTAGCTGGCGGAACTCCACGAGTGAGATTGGGTGTCACGTAGTAAGCATGAAGGCGAGACAAGGCGTGTATACTTGAAGGAGGTATAAGTTATGGGTAAGAAAGTTAAGAAGGCCGTGAAGAAAGTCACCAAGTCCGTTAAGAAAGTCGTTAAGGAAGGGGCTCGTAAGGTTAAACAGGTTGCTGGCGGTCTAGCTGGTCTGGCTGGTGGTACTGGTGAAGCACAGATGGTGGAAGTACCACAAGCTGCCGCACAGATTGTTGACGTACCTGAGaaa                                                                                                                                                                                   <  1:51119/301‑1 (MQ=255)`  
                                                                                                                                           `ttCCTAGCGAAACGCTTGAAGTACCTTGCGCAACTCCACGATGGCGATTGGGTGTCTCGTTGTAAGCATGAAGGCGCGACAATGCGTTTATACTTTAAGGAGGTATAAGTTATGGGTAAGAAAGTTAAGAAGGCCGTGAAGAGAGTCACCAAGTCCGTTAAGAAAGTCGTTAAGGAAGGGGCTCGTCCGGTTAAACAGGTTGCTGGCGGTCTAGCTGGTCTGGCTGGTGGTACTGGTGAAGCACAGATGGTGGAAGTACCACAAGCTGCCGCACAGATTGTTGACGTACCTGAGAAAGAgg                                                                                                                                                                               <  1:243026/301‑1 (MQ=255)`  
                                                                                                                                            `tCCTAGCGAAACGCTTTAATTACCTTGCGGAACTCCACGATTGCGATTGGGTGTCTCGTTGTAAGCATGAAGGCGAGACAATGCGTGTATACTTTAAGGAGGTATAAGTTATGGGTAAGAAAGTTAAGAAGGCCGTGAAGAAAGTCACCAAGTCCGTTAAGAAAGTCGTTAAGGAAGGGGCTCGTCCGGTTAAACAGGTTGCTGGCGGTCTAGCTGGTCTGGCTGGTGGTACTGGTGAAGCACAGATGGTGGAAGTACCACAAGCTGCCGCACAGATTGTTGACGTACCTGAGaactgtct                                                                                                                                                                              >  1:298950/1‑295 (MQ=255)`  
                                                                                                                                            `tCCTAGCGAAACGCTTTAAGTACCTTGCGGAACGCCACGATTGCGATTGGGTGTCTCGTTGTAAGCATGAAGGCGAGACAATGCGTGTATACTTTAAGGAGGTATAAGTTATGGGTAAGAAAGTTAAGAAGGCCGTGAAGAAAGTCACCAAGTCCGTTAAGAAAGTCGTTAAGGAAGGGGCTCGTCCGGTTAAACAGGTTGCTGGCGGTCTAGCTGGTCTGGCTGGTGGTACTGGTGAAGCACAGATGGTGGAAGTACCACAAGCTGCCGCACAGATTGTTGACGTACCTGAGAAAGAGGt                                                                                                                                                                              <  1:262567/301‑1 (MQ=255)`  
                                                                                                                                                  `gagacagCTTTAAGTACCTTGCGGAACTCCACGATTGCGATTGGGTGTCTCGTTGTAAGCATGAAGGCGAGAAAATGCGTGTATACTTTAAGGAGGTATAAGTTATGGGTAAGAAAGTTAAGAAGGCCGTGAAGAAAGTCACCAAGTCCGTTAAGAAAGTCGTTAAGGAAGGGGCTCGTCCGGTTAAACAGGTTGCTGGAGGTCTAGCTGGTCTGGCTGGTGGTACTGGTGAAGCACAGATGGTGGAAGTACCACAAGCTGCCGCACAGATTGTTGACGTACCTGAGAAAGAGGTTTCCAc                                                                                                                                                                        <  1:100031/295‑1 (MQ=255)`  
                                                                                                                                                    `aaaCGCTTTAAGTACCTTGCGGAACTCCACGATTGCGATTGGGTGTTTCGTTGTAAGCATGAAGGCGAGACAATGCGTGTATACTTTAAGGAGGTATAAGTTATGGGTAAGAAAGTGAAGAAGGCCGTGAAGAAAGTCACCAAGTCCGTTAAGAAAGTCGTTAAGGAAGGGGCTCGTCCGGTTAAACAGGTTGCTGGCGGTCTAGCTGGTCTGGCTGGTGGTACTGGTGAAGCACAGATGGTGGAAGTACCACAAGCTGCCGCACAGATTGTTGACGTACCTGAGAAAGAGGTTTCCACTg                                                                                                                                                                      <  1:116572/301‑1 (MQ=255)`  
                                                                                                                                                         `cTTTAAGTACCTTGCGGAACTCCACGATTGCGATTGGGTGTCTCGTTGTAAGCATGAAGGCGAGACAATGCGTGTATACTTTAAGGAGGTATAAGTTATGGGTAAGAAAGTTAAGAAGGCCGTGAAGAAAGTCACCAAGTCCGTTAAGAAAGTCGTTAAGGAAGGGGCTCGTCCGGTTAAACAGGTTGCTGGCGGTCTAGCTGGTCTGGCTGGTGGTACTGGTGAAGCACAGATGGTGGAAGTACCACAAGCTGCCGCACAGATTGTTGACGTACCTGAGAAAGAGGTTTCCAcctgtctc                                                                                                                                                                 >  1:105337/1‑294 (MQ=255)`  
                                                                                                                                                         `cTTTAAGTACCTTGCGGAACTCCACGATTGCGATTGGGTGTCTCGTTGTAAGCATGAAGGCGAGACAATGCATGTATACTTTAAGGAGGTATAAGTTATGGGTAAGAAAGTTAAGAAGGCCGTGAAGAAAGTCACCAAGTCCGTTAAGAAAGTCGTTAAGGAAGGGGCTCGTCCGGTTAAACAGGTTGCTGGCGGTCTAGCTGGTCTGGCTGGTGGTACTGGTGAAGCACAGATGGTGGAAGTATCACAAGCTGCCGCACAGATTGTTGACGTACCTGAGAAAGAGGTTTCCACTGAGTAc                                                                                                                                                                 >  1:174030/1‑301 (MQ=255)`  
                                                                                                                                                                `tACCTTGCGGTACTCCACGATTGCGATTTGGTGTCTCGTTGTAAGCATGAAGGCGAGACAATGCTTTTATACTTTAAGGATGTATAAGTTATGGGTAAGAAAGTTAATAAGGCCGTGAAGAAAGTCACCAAGTCCGTTAAGAAAGTCGTTAAGGAAGGTGCTCGTCCGGTTAAACAGGTTGCTGGCGGTCTAGCTGGTCTGGCTGGTGGTACTGGTGAAGCACAGATGGTGGAAGTACCACAAGCTGCCGCACAGATTGTTGACGTACCTGAGAAAGAGGTTTCCACTGAGGACGACGcac                                                                                                                                                          <  1:74508/301‑1 (MQ=255)`  
                                                                                                                                                                   `cTTGTGGATCTCCACGATTGCGATTGGGTGTCTCGTTGTAAGCATGAAGGCGAGACAATGCGTGTATACTTTAAGGAGGTATAAGTTATGGGTAAGAAAGTTAAGAAGGCCGTGAAGAAAGTCACCAAGTCCGTTAAGAAAGTCGTTAAGGAAGGGGCTCGTCCGGTTAAACAGGTTGCTGGCGGTCTAGCTGGTCTGGCTGGTGGTACTGGTGAAGCACAGATGGTGGAAGTACCACAAGCTGCCGCACAGATTGTTGACGTACCTGAGAAAGAGGTTTccctgtctcttatacacatct                                                                                                                                                       >  1:200971/1‑282 (MQ=255)`  
                                                                                                                                                                    `ttGCGGAACGCAACGATTGCGATTGGGGGTATTGTTGTAAACAGGAAGGCGAGAAAATGCGTGTATACTTAAAGGAGGTATAAGTGATGGGTAAGAAAGTTAAGAAGGCCGGGAAGAAAGTCACCAAGTCCGTTAAGAAAGTCGTTAAGGAAGAGGCTCGTCCGGTTAAACAGGTTGCTGGCGGTCTAGCTGGTCTGGCTGGTGGTACTGGTGAAGCACAGATGGTGGAAGTACCACAAGCTGCCGCACAGATTGTTGACGTACCTGAGAAAGAGGTTTCCACTGAGGACGAAGCacagac                                                                                                                                                      <  1:286616/301‑1 (MQ=255)`  
                                                                                                                                                                           `aCTCCACGATGGCGATGGGGTGTGTAGTTGTAAGAATGAAGGGGAGAAAATGCGTGTATACTTTAAGGAGGTATAAGTTATGGGTAAGAAAGTGAAGAAGGACGTGAAGAAAGTCACCAAGTCCGGTAAGAAAGTCGTTAAGGAAGGGGCTCTTCCGGTTAAACAGGTTGCTGGCGGTCTAGCTGGTCTGGCTGGTGGTACTGGTGAAGCACAGATGGTGGAAGTACCACAAGCTGCCGCACAGATTGTTGACGTACCTGAGAAAGAGGTTTCCACTGAGGACGAAGCACAGACAGAAAGc                                                                                                                                               <  1:4131/301‑1 (MQ=255)`  
                                                                                                                                                                              `ccACGATTGCGATTGGGTGTCTCGTTGTAAGCATGAAGGCGAGACAATGCGTGTATACTTTAAGGAGGTATAAGTTATGGGTAAGAAAGTTAAGAAGGCCGTGAAGAAAGTCACCAAGTCCGTTAAGAAAGTCGTTAACGAAGGGGCTCGTCCGGTTAAACAGGTTGCTGGCGGTCTAGCTGGTCTGGCTGGTGGTACTGGTGAAGCACAGATGGTGGAAGTACCACAAGCTGCCGCACAGATTGTTGACGTACCTGAGAAAGAGGTTTCCACTGAGGACGAAGCACAGCCCGAAAGCGGa                                                                                                                                            >  1:139512/1‑301 (MQ=255)`  
                                                                                                                                                                                         `attgGGTGTCTCGTTGTAAGCATGAAGGCGAGACAATGCGTGTATACTTTAAGTAGGTATAAGTTATGGGTAAGAAAGTTAAGAAGGCCGTGAAGAAAGTCACCAAGTCCGTTAAGAAAGTCGTTAAGGAAGGGGCTCGTCCGGTTAAACAGGTTGCTGGCGGTCTAGCTGGTCTGGCTGGTGGTACTGGTGAAGCACAGATGGTGGAAGTACCACAAGCTGCCGCACAGATTGTTGACGTACCTGAGAAAGAGGTTTCCACTGAGGACGAAGCACAGACAGAAAGCGGACGCAAGAAAGc                                                                                                                                 <  1:6965/301‑1 (MQ=255)`  
                                                                                                                                                                                            `gggTGTCTCGTTGTAAGCATGAAGGCGAGACAATGCGTGTATACTTTAAGGAGGTATAAGTTATGGGTAAGAAAGTTAAGAAGGCCGTGAAGAAAGTCACCAAGTCCGTTAAGAAAGTCGTTAAGGAAGGGGCTCGTCCGGTTAAACAGGTTGCTGGCGGTCTAGCTGGTCTGGCTGGTGGTACTGGTGAAGCACAGATGGTGGAAGTACCACAAGCTGCCGCACAGATTGTTGACGTACCTGAGAAAGAGGTTTCCACTGAGGACGAAGCACAGACAGAAAGCGGACGCAAGAAAGCTCg                                                                                                                              >  1:289405/1‑301 (MQ=255)`  
                                                                                                                                                                                                `gtCTCGTTGTAAGCATGAAGGCGAGACAATGCGTGTATACTTTAAGGAGGTATAAGTTATGGGTAAGAAAGTTAAGAAGGCCGTGAAGAAAGTCACCAAGTCCGTTAAGAAAGTCGTTAAGGAAGGGGCTCGTCCGGTTAAACAGGTTGCTGGCGGTCTAGCTGGTCTGGCTGGTGGTACTGGTGAAGCACAGATGGTGGAAGTACCACAAGCTGCCGCACAGATGGTTGACGTCCCGGAGAAAGAGGTTTCCACTGAGGACGAAGCACAGAAAGAAGGCGGACGCAAGAAAGCTCGTGCt                                                                                                                          >  1:127958/1‑301 (MQ=255)`  
                                                                                                                                                                                                    `cGTTGTAAGCATGGAGGCGAGACAATGCGTGTATACTTTAAGGAGGTATAAGTTATGGGTAAGAAAGTTAAGAAGGCCGTGAAGAAAGTCACCAAGTCCGTTAAGAAAGTCGTTAAGGAAGGGGCTCGTCCGGTTAAACAGGTTGCTGGCGGTCTAGCTGGTCTGGCTGGTGGTACTGGTGAAGCACAGATGGTGGAAGTACCACAAGCTGCCGCACAGATTGTTGACGTACCTGAGAAAGAGGTTTCCACTGAGGACGAAGCACAGACAGAAAGCGGACGCAAGAAAGCTCGTGCTGGCg                                                                                                                      >  1:82961/1‑301 (MQ=255)`  
                                                                                                                                                                                                     `gTTGTAAGCATGAAGGCGAGACAATGCGTGTATACTTTAAGGAGGTATAAGTTATGGGTAAGAAAGTTAAGAAGGCCGTGAAGAAAGTCACCAAGTCCGTTAAGAAAGTCGTTAAGGAAGGGGCTCGTCCGGTTAAACAGGTTGCTGGCGGTCTAGCTGGTCTGGCTGGTGGTACTGGTGAAGCACAGATGGTGGAAGTACCACAAGCTGCCGCACAGATTGTTGACGTACCTGAGAAAGAGGTTTCCACTGAGGACGAAGCACAGACAGAAAGCGGACGCAAGAAAGCTCGTGCTGGCgg                                                                                                                     >  1:330265/1‑301 (MQ=255)`  
                                                                                                                                                                                                      `ttGTAAGCATGAAGGCGAGACAATGCGTGTATACTTTAAGGAGGTATAAGTTATGGGTAAGAAAGTTAAGAAGGCCGTGAAGAAAGTAACCAAGTCCGTTAAGAAAGTCGTTAAGGAAGGGGCTCGTCCGGTTAAACAGGTTGCTGGCGGTCTAGCTGGTCTGGCTGGTGGTACTGGTGAAGCACAGATGGTGGAAGTACCACAAGCTGCCGCACAGATTGTTGACGTACCTGAGCAAGAGGTTTCCACTGAGGACGAAGCACAGACAGAACACGGACGCAAGAAAGCTCGTGCTGGCGGt                                                                                                                    >  1:227496/1‑301 (MQ=255)`  
                                                                                                                                                                                                          `aaGCATGAAGGCGAGACAATGCGTGTATACTTTAAGGAAGTATAAGTTATGGGTAAGAAAGTTAAGAAGGCCGTGAAGAAAGTCACCAAGTCCGTTAAGAAAGTCGTTAAGGAAGGGGCTCGTCCGGTTAAACAGGTTGCTGGCGGTCTAGCTGGTCTGGCTGGTGGTACTGGTGAAGCACAGATGGTGGAAGTACCACAAGCTGCCGCACAGATTGTTGACGTACCTGAGAAAGAGGTTTACACTGAGGACGAAGCACAGACAGAAAGCGGACGCAAGAAAGCTCGTGCTGGCGGTAAGa                                                                                                                >  1:76991/1‑301 (MQ=255)`  
                                                                                                                                                                                                           `ggCATTAAGGCGAGACAAGGCGTGTATACTTGAAGGAGGTATAAGTTATGGGTAAGAAAGTTAAGAAGGCCGTGAAGAAAGTCACCAAGTC‑‑‑‑‑‑‑‑‑‑‑‑CGTTAAGGAAGGGGCTCGTCCGGTTAAACAGGGTGCTGGCGGTCTAGCTGGTCTGGCTGGTGGTACTGGTGAAGCACAGATGGTGGAAGTACCACAAGCTGCCGCACAAATTGTTGACGTACCTGAGAAAGAGGTTTCCACTGAGGACGAAGCACAGACAGAAAGCGGACGCAAGAAAGCTCGTGCTGGCGGTAAGAAATCCTTGAgtgt                                                                                                   <  1:332728/300‑1 (MQ=255)`  
                                                                                                                                                                                                            `gCATGAAGGCGAGACAATGCGGGGATACTTTAAGGAGGTATAAGGTAGGGGTAAGAAAGTTAAGAAGGCCGTGAAGAAAGTCACCAAGTCCGTTAAGAAAGTCGTTAAGGAAGGGGCTCGTCCGGTTAAACAGGTTGCTGGCGGTCTAGCTGGTCTGGCTGGTGGTACTGGTGAAGCACAGATGGTGGAAGTACCACAAGCTGCCGCACAGATTGTTGACGTACCTGAGAAAGAGGTTTCCACTGAGGACGAAGCACAGACAGAAAGCGGACGCAAGAAAGCTCGTGCTGGAGGTAAGaaa                                                                                                              <  1:6091/301‑1 (MQ=255)`  
                                                                                                                                                                                                                    `gCGAGACAATGCGTGTATACTTTAAGGAGGTATAAGTTATGGGTAAGAAAGTTAAGAAGGCCGTGAAGAAAGTCACCAAGTCCGTTAAGAAAGTCGTTAAGGAAGGGGCTCGTCCGGTTAAACAGGTTGCTGGCGGTCTAGCTGGTCTGGCTGGTGGTACTGGTGAAGCACAGATGGTGGAAGTACCACAAGCTGCCGCACAGATTGTTGACGTACCTGAGAAAGAGGTTTCCACTGAGGACGAAGCACAGACAGAAAGCGGACGCAAGAAAGCTCGTGCTGGCGGTAAGAAATCCTTGAg                                                                                                      <  1:58511/301‑1 (MQ=255)`  
                                                                                                                                                                                                                      `gagaCAATGCGTGTATACTTTAAGGAGGTATAAGTTATGGGTAAGAAAGTTAAGAAGGCCGTGAAGAAAGTCACCAAGTCCGTTAAGAAAGTCGTTAAGGAAGGGGCTCGTCCGGTTAAACAGGTTGCTGGCGGTCTAGCTGGTCTGGCTGGTGGTACTGGTGAAGCACAGATGGTGGAAGTACCACAAGCTGCCGCACAGATTGTTGACGTACCTGAGAAAGAGGTTTCCACTGAGGACGAAGCACAGACAGAAAGCGGACGCAAGAAAGCTCGTGCTGGCGGTAAGAAATCCtgtctct                                                                                                    >  1:37786/1‑295 (MQ=255)`  
                                                                                                                                                                                                                       `agaCAATGCGTGTATACTTTAAGGAGGTATAAGTTATGGGTAAGAAAGTTAAGAAGGCCGTGAAGAAAGTCACCAAGTCCGTTAAGAAAGTCGTTAAGGAAGGGGCTCGTCCGGTTAAACAGGTTGCTGGCGGTCTA‑‑‑‑‑‑‑‑‑GCTGGTGGTACTGGTGAAGCACAGATGGTGGAAGTACCACAAGCTGCCGCACAGATTGTTGACGTACCTGAGAAAGAGGTTTCCACTGAGGACGAAGCACAGACAGAAAGCGGACGCAAGAAAGCTCGTGCTGGCGGGAAGAAATCCTTGAGTGTAGCACGTAg                                                                                          >  1:312114/1‑301 (MQ=255)`  
                                                                                                                                                                                                                         `aCAATGCGTGTATACTTTAAGGAGGTATAAGTTATGGGTAAGAAAGTTAAGAAGGCCGTGAAGAAAGTCACCAAGTCCGTTAAGAAAGTCGTTAAGGAAGGGGCTCGTCCGGTTAAACAGGTTGCTGGCGGTCTAGCTGGTCTGGCTGGTGGTACTGGTGAAGCACAGATGGTGGAAGTACCACAAGCTGCCGCACAGATTGTTGACGTACCTGAGAAAGAGGTTTCCACTGAGGACGAAGCACAGACAGAAAGCTGACGCAAGAGAGCTCATGCTGGCGGTAAGaaactgcctattataa                                                                                                 >  1:315296/1‑288 (MQ=255)`  
                                                                                                                                                                                                                            `aTGCTTGTATACTTGAAGGAGGTATAAGTTATGGGTAAGAAAGTTAAGAAGGCCGTGAAGAAAGTCACCAAGTCCGTTAAGAAAGTCGTTAAGGAAGGGGCTCGTCCGGTTAAACAGGTTGCTGGCGGTCTAGCTGGTCTGGCTGGTTGTACTGGTGAAGCACAGATGGTGGAAGTACCACAAGCTGCCGCACAGATTGTTGACGTACCTGAGAAAGAGGTTTCCACTGAGGACGAAGCACAGACAGAAAGCGGACGCAAGAAAGCTCGTGCTGGCGGTAAGAAATCCTTGAGTGTAGccc                                                                                              <  1:92693/301‑1 (MQ=255)`  
                                                                                                                                                                                                                              `gCGTGTATACTTTAAGGAGGTATAAGTTATGGGTAAGAAAGTTAAGAAGGCCGTGAAGAAAGTCACCAAGTCCGTTAAGAAAGTCGTTAAGGAAGGGGATCGTCCGGTTAAACAGGTTGCTGGCGGTCTAGCTGGTCTGGCTGGTGATCCTGGTGAAGCACAGATGGTGGGAGTACCACAAGCTGCCGCACAGATTGTTGACGTACCTGAGAAAGAGGTTTCCACTGAGGACGAAGCACAGACAGAAAGCGGACGCAAGAAAGCTCGTGCTGGCGGTAAGAAATCCTTGAGTGTAGCACgg                                                                                            >  1:65208/1‑300 (MQ=255)`  
                                                                                                                                                                                                                               `cGTGTATACTTTAAGGAGGTATAAGTTATGGGTAAGAAAGTTAAGAAGGCCGTGAAGAAAGTCACCAAGTCCGTTAAGAAAGTCGTTAAGGAAGGGGCTCGTCCGGTTAAACAGGTTGCTGGCGGTCTAGCTGGTCTGGCTGGTGGTACTGGTGAAGCACAGATGGTGGAAGTACCGCAAGCTGTCGCACAGATTGTTGACGTACCTGAGAAAGAGGTTTCCACTGAGGACGAAGCACAGACAGAAAGCGGACGCAAGAAAGCTCGTGCTGGCGGTAAGAAATCCTTGAGTGTAGCCCGTa                                                                                           >  1:233527/1‑301 (MQ=255)`  
                                                                                                                                                                                                                                 `tgtATACTTTAAGGAGGTATAAGTTATGGGTAAGAAAGTTAAGAAGGCCGTGAAGAAAGTCACCAAGTCCGTTAAGAAAGTCGTTAAGGAAGGGGCTCGTCCGGTTAAACAGGTTGCTGGCGGTCTAGCTGGTCTGGCTGGTGGTACTGGTGAAGCACAGATGGTGGAAGTACCACAAGCTGCCGCACAGATTGTTGACGTACCTGAGAAAGAGGTTTCCACTGAGGACGAAGCACAGACAGAAAGCGGACGCAAGAAAGCTCGTGCTGGCGGTAAGAAATCCTTGAGTGTAGCCCGTAGc                                                                                         <  1:308825/301‑1 (MQ=255)`  
                                                                                                                                                                                                                                 `ggtATACTATAAGGAGGTATAAGTTATGGGTAAGAAAGGTAAGAAGGCCGTGAAGAAAGTCACCAAGTCCGTTAAGAAAGTCGTTAAGGAAGGGGCTCGTCCGGTTAAACAGGTTGCTGGCGGTCTAGCTGGTCTGGCTGGTGGTACTGGTGAAGCACAGATGGTGGAAGTACCACAAGCTGCCGCACAGATTGTTGACGTACCTGAGAAAGAGGTTTCCACTGAGGACGAAGCACAGACAGAAAGCGGACGCAAGAAAGCTCGTGCTGTCGGTAAGAAATCCTTGAGTGTAGCCCGTAGc                                                                                         <  1:135288/300‑1 (MQ=255)`  
                                                                                                                                                                                                                                   `tataCTTTAAGGAGGTATAAGTTATGGGTAAGAAAGTTAAGAAGGCAGTGAAGAAAGTCACCAAGTCCGTTAAGAAAGTCGTTAAGGAAGGGGCTCGTCCGGTTAAACAGGTTGCTGGCGGTCTAGCTGGTCTGGCTGGTGGTACTGGTGAAGCACAGATGGTGGAAGTACCACAAGCTGCCGCACAGATTGTTGACGTACCTGAGAAAGAGGTTTCCACTGAGGACGAAGCACAGACAGAAAGCGGACGCAAGAAAGCTCGTGCTGGCGGTAAGAAATCCTTGAGTGTAGCCCGTAGCTc                                                                                       <  1:230714/301‑1 (MQ=255)`  
                                                                                                                                                                                                                                   `tataCTGTAAGGAGGTATAAGTTATGGGTAAGAAAGTTAAGAAGGCCGTGAAGAAAGTCACCAAGTCCGTTAAGAAAGTCGTTAAGGAAGGGGCTCGTCCGGTTAAACAGGTTGCTGGCGGTCTAGCTGGTCTGGCTGGTGGTACTGGTGAAGCACAGATGGTGGAAGTACCACAAGCTGCCGCACAGATTGTTGACGTACCTGAGAAAGAGGTTTCCACTGAGGACGAAGCACAGACAGAAAGCGGACGCAAGAAAGCTCGTGCTGGCGGTAAGAAATCCTTGAGTGTAGCCCGTAGCTc                                                                                       <  1:187791/301‑1 (MQ=255)`  
                                                                                                                                                                                                                                       `cTTTAAGGAGGTATAAGTTATGGGTAAGAAAGTTAAGAAGGCCGTGAAGAAAGTCACCAAGTCCGTTAAGAAAGTCGTTAAGGAAGGGGCTCGTCCGGTTAAACAGGTTGCTGGCGGTCTAGCTGGTCTGGCTGGTAATACTGGTGAAGCACAGATGGTGGAAGTACCACAAGCTGCCGCACAGATTGTTGACGTACCTGAGAAAGAGGTTTCCACTGAGGACGAAGCACAGACAGAAAGCGGACGCAAGAAAGCTCGTGCTGGCGGTAAGAAATCCTTGAGTGTAGCCCGTAGCTCCGGt                                                                                   >  1:146812/1‑301 (MQ=255)`  
                                                                                                                                                                                                                                               `aggTATAAGTTATGGGTAAGAAAGTTAAGAAGGCCGTGAAGAAAGTCACCAAGTCCGTTAAGAAAGTCGTTAAGGAAGGGGCTCGTCCGGTTAAACAGGTTGCTGGCGGTCTAGCTGGTCTGGCTGGTGGTACTGGTGAAGCACAGATGGTGGAAGTACCACAAGCTGCCGCACAGATTGTTGACGTACCTGAGAAAGAGGTTTCCACTGAGGACGAAGCACAGACAGAAAGCGGACGCAAGAAAGCTCGTGCTGGCGGTAAGAAATCCTTGAGTGTAGCCCGTAGCTCCGGTGGCGGTAt                                                                           >  1:254250/1‑301 (MQ=255)`  
                                                                                                                                                                                                                                                `ggTATAAGTTATGGGTAAGAAAGTTAAGAAGGCCGTGAAGAAAGTCACCAAGTCCGTTAAGAAAGTCGTTAAGGAAGGGGCTCGTCCGGTTAAACAGGTTGCTGGCGGTCTAGCTGGTCTGGCTGGTGGTACTGGTGAAGCACAGATGGTGGAAGTACCACAAGCTGCCGCACAGATTGTTGACGTACCTGAGAAAGAGGTTTCCACTGAGGACGAAGCACAGACAGAAAGCGGACGCAAGACAGCTCGTGCTGGCGGTAAGAAATCCTTGAGTGTAGCCCGTAGCAACGGTGGCGGTATc                                                                          >  1:131611/1‑301 (MQ=255)`  
                                                                                                                                                                                                                                                `ggTATAAGTTATGGGTAAGAAAGTTAAGAAGGCCGTGAAGAAAGTCACCAAGTCCGTTAAGAAAGTCGTTAAGGAAGGGGCTCGTCCGGTTAAACAGGTTGCTGGCGGTCTAGCTGGTCTGGCTGGTGGTACTGGTGAAGCACAGATGGTGGAAGTACCACAAGCTGCCGCACAGATTGTTGACGTACCTGAGAAAGAGGTTTCCACTGAGGACGAAGCACAGACAGAAAGCGGACGCAAGAAAGCTCGTGCTGGCGGTAAGAAATCCTTGAGTGTAGCCCGTAGCTCCGGTGGCGGTATc                                                                          >  1:326824/1‑301 (MQ=255)`  
                                                                                                                                                                                                                                                `ggTATAAGTTATGGGTAAGAAAGTTAAGAAGGCCGTGAAGAAAGTCACCAAGTCCGTTAAGAAAGTCGTTAAGGAAGGGGCTCGTCCGGTTAAACAGGTTGCTGGCGGTCTAGCTGGTCTGGCTGGTGGTACTGGTGAAGCACAGATGGTGGAAGTACCACAAGCTGCCGCACAGATTGTTGACGTACCTGAGAAAGAGGTTTCCACTGAGGACGAAGCACAGACAGAAAGCGGACGCAAGAAAGCTCGTGCTGGCGGTAAGAAATCCTTGAGTGTAGCCCGTAGCTCCGGTGGCGGTATc                                                                          >  1:166228/1‑301 (MQ=255)`  
                                                                                                                                                                                                                                                `ggTATAAGTTATGGGTAAGAAAGTTAAGAAGGCCGTGAAGAAAGTCACCAAGTCCGTTAAGAAAGTCGTTAAGGAAGGGGCTCGTCCGGTTAAACAGGTTGCTGGCGGTCTAGCTGGTCTGGCTGGTGGTACTGGTGAAGCACAGATGGTGGAAGTACCACAAGCTGCCGCACAGATTGTTGACGTACCTGAGAAAGAGGTTTCCACTGAGGACGAAGCACAGACAGAAAGCGGACGCAAGAAAGCTCGTGCTGGCGGTAAGAAATCCTTAAGTGTAGCCCGTAGCTCCGGTGGCGGTATc                                                                          >  1:112482/1‑301 (MQ=255)`  
                                                                                                                                                                                                                                                 `gTATAAGTTATGGGTAAGAAAGTTAAGAAGGCCGTGAAGAAAGTCACCAAGTCCGTTAAGAAAGTCGTTAAGGAAGGGGCTCGTCCGGTTAAACAGGTTGCTGGCGGTCTAGCTGGTCTGGCTGGTGGTACTGGTGAAGCACAGATGGTGGAAGTACCACAAGCTGCCGCACAGATTGTTGACGTACCTGAGAAAGAGGTTTCCACTGAGGACGAAGCACAGACAGAAAGCGGACGCAAGAAAGCTCGTGCTGGCGGTAAGAAATCCTTGAGTGTAGCCCGTAGCTCCGGTGGCGGTATCa                                                                         >  1:164000/1‑301 (MQ=255)`  
                                                                                                                                                                                                                                                    `tgaGTTAAGGGTAAGAAAGTTAAGAAGGCCGTGAAGAAAGTCACCAAGGCCGTTAAGAAAGTCGTTAAGGAAGGGGCTAGTCCGGTTAAACAGGGTGCTGGCGGTCTAGCTGGTCTGGCTGGTGGTACTGGTGAAGCACAGATGGTGGAAGTACCACAAGCTGCCGCACAGATTGTTGACGTACCTGAGAAAGAGGTTTCCACTGAGGACGAAGCACAGACAGAAAGCGGACGCAAGAAAGCTCGTGCTGGCGGTAAGAAATCCTTGAGTGTAGACCGTAGCTCCGGTGGCGGTATCAACa                                                                      <  1:179198/299‑1 (MQ=255)`  
                                                                                                                                                                                                                                                     `aaGTTATGGGTAAGAAAGTTAAGAAGGCCGTGAAGAAAGTCACCAAGTCCGTTAAGAAAGTCGTTAAGGAAGGGGCTCGTCCGGTTAAACAGGTTGCTGGCGGTCTAGCTGGTCTGGCTGGTGGTACTGGTGAAGCACAGATGGTGGAAGTACCACAAGCTGCCGCACAGATTGTTGACGTACCTGAGAAAGAGGTTTCCACTGAGGACGAAGCACAGACAGAAAGCGGACGCAAGAAAGCTCGTGCTGGCGGTAAGAAATCCTTGAGTGTAGCCCGTAGCTCCGGTGGCGGTATCAACAt                                                                     >  1:310745/1‑301 (MQ=255)`  
                                                                                                                                                                                                                                                      `aGTTATGGGTAAGAAAGTTAAGAAGGCCGTGAAGAAAGTCACCAAGTCCGTTAAGAAAGTCGTTAAGGAAGGGGCTCGTCCGGTTAAACAGGTTGCTGGCGGTCTAGCTGGTCTGGCTGGTGGTACTGGTGAAGCACAGATGGTGGAAGTACCACAAGCTGCCGCACAGATTGTTGACGTACCTGAGAAAGAGGTTTCCACTGAGGACGAAGCACAGACAGAAAGCGGACGCAAGAAAGCTCGTGCTGGCGGTAAGAAATCCTTGAGTGTAGCCCGTAGCTCCGGTGGCGGTATCAACAtt                                                                    >  1:340932/1‑301 (MQ=255)`  
                                                                                                                                                                                                                                                           `tGGGTAAGAAAGTTAAGAAGGCCGTGAAGAAAGTCACCAAGTCCGTTAAGAAAGTCGTTAAGGAAGGGGCTCGTCCGGTTAAACAGGTTGCTGGCGGTCTAGCTGGTCTGGCTGGTGGTACTGGTGAAGCACAGATGGTGGAAGTACCAAAAGCTGCCGCACAGATTGTTGACGTACCTGAGAAAGAGGTTTCCACTGAGGACGAAGCACAGACAGAAAGCGGACGCAAGAAAGCTCGTGCTGGCGGTAAGAAATCCTTGAGTGGAGCCCGTAGCTCCGGTGGCGGTATCAACATTTAATc                                                               <  1:144410/301‑1 (MQ=255)`  
                                                                                                                                                                                                                                                           `ggggTAAGAAAGTTAAGAAGGCCGTGAAGAAAGTCACCAAGTCCGTTAAGAAAGTCGTTAAGGAAGGGGCTCGTCCGGTAAAACAGGTTGCTGGCGGTCTAGCTGGTCTGGCTGGTGGTACTGGTGAAGCACAGATGGTGGAAGTACCACAAGCTGCCGCACAGATTGTTGACGTACCTGAGAAAGAGGTTTCCACTGAGGACGAAGCACAGACAGAAAGCGGACGCAAGAAAGCTCGTGCTGGCGGTAAGAAATCCTTGAGTGTAGCCCGTAGCTCCGGTGGCGGTATCAACATTTAATc                                                               <  1:303505/300‑1 (MQ=255)`  
                                                                                                                                                                                                                                                           `ataagagacaggTTAAGAAGGCCGTGAAGAAAGTCACCAAGTCCGTTAAGAAAGTCGTTAAGGAAGGGGCTCGTCCGGTTAAACAGGTTGCTGGCGGTCTAGCTGGTCTGGCTGGTGGTACTGGTGAAGCACAGATGGTGGAAGTACCACAAGCTGCCGCACAGATTGTTGACGTACCTGAGAAAGAGGTTTCCACTGAGGACGAAGCACAGACAGAAAGCGGACGCAAGAAAGCTCGTGCTGGCGGTAAGAAATCCTTGAGTGTAGCCCGTAGCTCCGGTGGCGGTATCAACATTTAATc                                                               <  1:62790/290‑1 (MQ=255)`  
                                                                                                                                                                                                                                                            `gggTAAGAAAGTTAAGAAGGCCGTGAAGAAAGTCACCAAGTCCGTTAAGAAAGTCGTTAAGGAAGGGGCTCGTCCGGTTAAACAGGTTGCTGGCGGTCTAGCTGGTCTGGCTGGTGGTACTGGTGAAGCACAGATGGTGGAAGTACCACAAGCTGCCGCACAGATTGTTGACGTACCTGAGAAAGAGGTTTCCACTGAGGACGAAGCACAGACAGAAAGCGGACGCAAGAAAGCTCGTGCTGGCGGTAAGAAATCCTTGAGTGTAGCCCGTAGCTCCGGTGGCGGTATCAACATTTAATCa                                                              >  1:283968/1‑301 (MQ=255)`  
                                                                                                                                                                                                                                                              `gTAAGAAAGTTAAGAAGGCCGTGAAGAAAGTCCACAAGTCCGTTGAGTAAGTCGTTAAGGAAGCGGCCCGTCCGATTAAACAGGTTGCTGGCGGTCTAGCTGTACTGGCTGATGGTACTGGTGAAGCTCAGATGGTGGCAGTACCACAAGCTGCCGCTCAGATTGTTGACGTACCTTACCACGAGGTTTCCACTGAGGACGAATCACAGCCAGAACGCGGACGCAAGAAAGTTCGTCCTGCCGGTAAGTAATTCTTGAGTGTAGCCCATATCTCCGGTGTCGGCATCAACATTTCATCagg                                                            >  1:215901/1‑301 (MQ=255)`  
                                                                                                                                                                                                                                                                `agacaggTTAAGAAGGCCGTGAAGAAAGTCACCAAGTCCGTTAAGAAAGTCGTTAAGGAAGGGGCTCGTCCGGTTAAACAGGTTGCTGGCGGTCTAGCTGGTCTGGCTGGTGGTACTGGTGAAGCACAGATGGTGGAAGTACCACAAGCTGCCGCACAGATTGTTGACGTACCTGAGAAAGAGGTTTCCACTGAGGGCGAAGCACAGACAGAAAGCGGACGCAAGAAAGCTCGTGCTGGCGGTAAGAAATCCTTGAGTGTAGCCCGTAGCTCCGGTGGCGGTATCAACATTTAATCaggag                                                          <  1:203888/295‑1 (MQ=255)`  
                                                                                                                                                                                                                                                                      `gTTAAGAAGGCCGTGAAGAAAGTCACCAAGTCCGTTAAGAAAGTCGTTAAGGAAGGGGCTCGTCCGGTTAAACAGGTTGCTGGCGGTCTAGCTGGTCTGGCTGGTGGTACTGGTGAAGCACAGATGGTGGAAGTACCACAAGCTGCCGCACAGATTGTTGACGTACCTGAGAAAGAGGTTTCCACTGAGGACGAAGCACAGACAGAAAGCGGACGCAAGAAAGCTCGTGCTGGCGGTAAGAAATCCTTGAGTGTAGCCCGTAGCTCCGGTGGCGGTATCAACATTTAATCAGGAGGTTATc                                                    >  1:15736/1‑301 (MQ=255)`  
                                                                                                                                                                                                                                                                      `gTTAAGAAGGCCGTGAAGAAAGTCACCAAGTCCGTTAAGAAAGTCGTTAAGGAAGGGGCTCGTCCGGTTAAACAGGTTGCTGGCGGTCTAGCTGGTCTGGCTGGTGGTACTGGTGAAGCACAGATGGTGGAAGTACCACAAGCTGCCGCACAGATTGTTGACGTACCTGAGAAAGAGGTTTCCACTGAGGACGAAGCACAGACAGAAAGCGGACGCAAGAAAGCTCGTGCTGGCAGTAAGAAATCCTTGAGTGTAGCCCGTAGCTCCGGTGACGGTATCAACATTTAATCAGGAGGGTATc                                                    >  1:81336/1‑301 (MQ=255)`  
                                                                                                                                                                                                                                                                           `acagGCCGTGAAGAAAGTCACCAAGTCCGGTAAGAAAGTCGTTAAGGAAGGGGCTCGTCCGGTTAAACAGGTTTCTGGCGGTCTAGCTGGTCTGGCTGGTGGTACTGGTGAAGCACAGATGGTGGAAGTACCACAAGCTGCCGCACAGATTGTTGACGTACCTGAGAAAGAGGTTTCCACTGAGGACGAAGCACAGACAGAAAGCGGACGCAAGAAAGCTCGTGCTGGCGGTAAGAAATCCTTGAGTGTAGCCCGTAGCTCCGGTGGCGGTATCAACATTTAATCAGGAGGTTATCGTGGa                                               <  1:102862/299‑1 (MQ=255)`  
                                                                                                                                                                                                                                                                               `gCCGTGAAGAAAGTCACCAAGTGCGTTAAGAAAGTCGTTAATGAAGGGGCTCGTCCGGTTAAACAGGTTGCTGGCGGTCTAGCTGGTCTGGCTGGTGGTACTGGTGAAGCACAGATGGTGGAAGTACCACAAGCTGCCGCACAGATTGTTGACGTACCTGAGAAAGAGGTTTCCACTGAGGACGAAGCACAGACAGAAAGCGGACGCAAGAAAGCTCGTGCTGGCGGTAAGAAATCCTTGAGTGTAGCCCGTAGCTCCGGTGGCGGTATCAACATTTAATCAGGAGGTTATCGTGGAAGAc                                           <  1:239607/301‑1 (MQ=255)`  
                                                                                                                                                                                                                                                                                         `aaGTCAGCAAGTCCGTTAAGAAAGTCGTTAAGGAAGGGGCTCGTCCGGTTAAACAGGTTGCTGGCGGTCTAGCTGGTCTGGCTGGTGGTACTGGTGAAGCACAGATGGTGGAAGTACCACAAGCTGCCGCACAGATTGTTGACGTACCTGAGAAAGAGGTTTCCACTGAGGACGAAGCACAGACAGAAAGCGGACGCAAGAAAGCTCGTGCTGGCGGTAAGAAATCCTTGAGTGTAGCCCGTAGCTCCGGTGGCGGTATCAACATTTAATCAGGAGGTTATCGTGGAAGACTGCATTGAAt                                 <  1:280082/301‑1 (MQ=255)`  
                                                                                                                                                                                                                                                                                         `aaGTCACCAAGGCCGTTAAGAAAGTCGTTAAGGAAGGGGCTCGTCCGGTTAAACAGGTTGCTGGCGGTCTAGCTGGTCTGGCTGGTGGTACTGGTGAAGCACAGATGGTGGAAGTACCACAAGCTGCCGCACAGATTGTTGACGTACCTGAGAAAGAGGTTTCCACTGAGGACGAAGCACAGACAGAAAGCGGACGCAAGAAAGCTCGTGCTGGCGGTAAGAAATCCTTGAGTGTAGCCCGTAGCTCCGGTGGCGGTATCAACATTTAATCAGGAGGTTATCGTGGAAGACTGCATTGAAt                                 <  1:190691/301‑1 (MQ=255)`  
                                                                                                                                                                                                                                                                                           `gTCACCAAGTCCGTTAAGAAAGTCGTTAAGGAAGGGGCTCGTCCGGTTAAACAGGTTGCTGGCGGCCTAGCTGGTCTGGCTGGTGGTACTGGTGAAGCACAGATGGGGGAAGTAACACAAGCTGCCGCACAGATTGTTGACGTACCTGCGAAAGAGGTTTCCACTGAGGACGAAGCACCGACAGAAAGCGGACGCAAGAAAGCTCGTGCTGGCGGGAAGAAATCATTGAGTGTAGCCCGGAGCTCCGGTGGCGGTATCAACATTTAATCAGGAGGGTATCGTGGAAGACTGCATTGAATgg                               >  1:306519/1‑301 (MQ=255)`  
                                                                                                                                                                                                                                                                                                `caggTCCGTGGAGAAAGTCGTTAAGGAAGGTGCTCGTCCGGTTAAACAGGTTGCTGGCGGGCTAGCTGGTCTGGCTGGTGGTACTGGTGAAGCACAGATGGTGGAAGTGCCTCAAGCTGCCGCACAGATTGTTGACGTACGTGAGAAAGAGGTTTCCACTGAGGACGAAGCACAGACAGAAAGCGGACGCAAGGAAGCTCGTGCTGGCGGTAAGAAATCCTTGAGTGTAGCCCGTAGCTCCGGTGGCGGTATCAACATTTAATCAGGAGGTTATCGTGGAAGACTGCATTGAATGGACCgg                          <  1:318619/298‑1 (MQ=255)`  
                                                                                                                                                                                                                                                                                                   `gTCCGTTAAGAAAGTCGTTAAGGAAGGGGCTCGTCAGGTTAAACAGGTTGCTGGCGGTGTAGCTGGTCTGGCTGGTGGTACTGGTGAAGCACAGATGGTGGAAGTACCACAAGCTGCCGCACAGATTGTTGACGTACCTGAGAAAGAGGTTTCCACTGAGGACGAAGCACAGACAGAAAGCGGACGCAAGAAAGCTCGTGCTGGCGGTAAGAAATCCTTGAGTGTAGCCCGTAGCTCCGGTGGCGGTATCAACATTTAATCAGGAGGTTATCGTGGAAGACTGCATTGAATGGACCGGAgg                       <  1:235952/301‑1 (MQ=255)`  
                                                                                                                                                                                                                                                                                                            `gAAAGTCGTTAAGGAAGGGGCTCGTCCGGTTAAACAGGTTTCTGGCGGTCTAGCTGGTCTGGCTGGTGGTACTGGTGAAGCACAGATGGTGGAAGTACCACAAGCTGCCGCACAGATTGTTGACGTACCTGAGAAAGAGGTTTCCACTGAGGACGAAGCACAGACAGAAAGCGGACGCAAGAAAGCTCGTGCTGGCGGTAAGAAATCCTTGAGTGTAGCCCGTAGCTCCGGTGGCGGTATCAACATTTAATCAGGAGGTTATCGTGGAAGACTGCATTGAATGGACCGGAGGTGTCAActc              <  1:175763/301‑1 (MQ=255)`  

                                                                                                                                                                                                                                                                                                            `|`                                                                                                                                                                                                                                                                                                                          
`GTACATTCTGGACTCTACCGGAAAACATGTAGCGTACTTCGCGTGGTGCGTAAGCTGTGACATTCACCACAAAGGAGACATTCTGGATGTAACGTCCGTTGTCATTAATCCTGAGGCAGACTCTAAGGGCTTACAGCGATTCCTAGCGAAACGCTTTAAGTACCTTGCGGAACTCCACGATTGCGATTGGGTGTCTCGTTGTAAGCATGAAGGCGAGACAATGCGTGTATACTTTAAGGAGGTATAAGTTATGGGTAAGAAAGTTAAGAAGGCCGTGAAGAAAGTCACCAAGTCCGTTAAGAAAGTCGTTAAGGAAGGGGCTCGTCCGGTTAAACAGGTTGCTGGCGGTCTAGCTGGTCTGGCTGGTGGTACTGGTGAAGCACAGATGGTGGAAGTACCACAAGCTGCCGCACAGATTGTTGACGTACCTGAGAAAGAGGTTTCCACTGAGGACGAAGCACAGACAGAAAGCGGACGCAAGAAAGCTCGTGCTGGCGGTAAGAAATCCTTGAGTGTAGCCCGTAGCTCCGGTGGCGGTATCAACATTTAATCAGGAGGTTATCGTGGAAGACTGCATTGAATGGACCGGAGGTGTCAACTCTAAGGGTTATGG  >  NC_001604.1/19285‑19897`

  

Alignment Legend

Aligned base mismatch/match (shaded by quality score): `ATCG/ATCG` `< 3 ≤` `ATCG/ATCG` `< 10 ≤` `ATCG/ATCG` `< 22 ≤` `ATCG/ATCG` `< 34 ≤` `ATCG/ATCG` `< 38 ≤` `ATCG/ATCG`

Unaligned base: `atcg`    Masked matching base: `atcg`    Alignment gap: `‑`    Deleted base: `‑`

Reads not counted as support for junction

read\_name Not counted due to insufficient overlap past the breakpoint.

read\_name Not counted due to not crossing MOB target site duplication.
